# A quantum spin-probe molecular microscope

**DOI:** 10.1038/ncomms12667

**Published:** 2016-10-11

**Authors:** V. S. Perunicic, C. D. Hill, L. T. Hall, L.C.L. Hollenberg

**Affiliations:** 1 Centre for Quantum Computation and Communication Technology, School of Physics, University of Melbourne, Melbourne, Victoria 3010, Australia; 2 School of Physics, University of Melbourne, Melbourne, Victoria 3010, Australia

## Abstract

Imaging the atomic structure of a single biomolecule is an important challenge in the physical biosciences. Whilst existing techniques all rely on averaging over large ensembles of molecules, the single-molecule realm remains unsolved. Here we present a protocol for 3D magnetic resonance imaging of a single molecule using a quantum spin probe acting simultaneously as the magnetic resonance sensor and source of magnetic field gradient. Signals corresponding to specific regions of the molecule's nuclear spin density are encoded on the quantum state of the probe, which is used to produce a 3D image of the molecular structure. Quantum simulations of the protocol applied to the rapamycin molecule (C_51_H_79_NO_13_) show that the hydrogen and carbon substructure can be imaged at the angstrom level using current spin-probe technology. With prospects for scaling to large molecules and/or fast dynamic conformation mapping using spin labels, this method provides a realistic pathway for single-molecule microscopy.

## Abstract (web summary)

Single spin defects can allow high-resolution sensing of molecules under an applied magnetic field. Here, the authors propose a protocol for three-dimensional magnetic resonance imaging with angstrom-level resolution exploiting the dipolar field of a spin qubit, such as a diamond nitrogen-vacancy.

## Graphical abstract

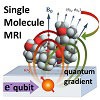

In conventional techniques used to determine biomolecular structure such as X-ray crystallography, nuclear magnetic resonance (NMR) spectroscopy or free electron laser femtosecond serial crystallography, large numbers of the target molecule are required to obtain sufficient signal. To obtain structural information at the single-molecule level, as close to *in situ* as possible, one requires a true molecular microscope. In this quest, the tantalising prospect of using quantum spin probes for the detection of nuclear spins in molecules has been a key driver of much of the research in quantum sensing using the nitrogen-vacancy (NV) centre in diamond^1^,^2^, with recent experimental demonstrations of internal and external nuclear spin detection under ambient conditions^3^,^4^,^5^,^6^,^7^,^8^,^9^,^10^,^11^,^12^,^13^. These breakthroughs have spurred a number of theoretical proposals for molecular level NMR, including methods for molecular detection based on intrinsic nuclear flipping rates^14^, Rabi driving induced resonant detection^15^, 1-D NMR spectroscopy^16^ and amplified long-range sensing of nuclear spins^17^. Of particular note are proposals heading towards molecular structure determination based on 2-D NMR-related spectroscopy^18^,^19^,^20^, and magnetic tip induced field gradient imaging^21^. Recently, a technique to use a NV-centre probe together with a Hamiltonian interrogation protocol has been proposed for nuclear localisation of individual spin labels based on the determination of their full hyperfine tensor in the weak field regime^22^. However, it is not clear whether these techniques can achieve structure determination with angstrom-level resolution for real-world size biomolecules required for biological applications. Highly stable external gradient fields, that are significantly stronger than presentably achievable, are required to sufficiency surpass the ∼70 G nm^−1^ threshold where gradient splitting becomes equal to the typical nuclear dipole–dipole coupling. While 2D NMR techniques may suffer from the same limitations of their ensemble counterparts in terms of the maximum molecule size for which the complex frequency spectra inversion can be carried out^23^,^24^,^25^,^26^,^27^.

In this paper, we present a method based on a quantum spin probe which acts as a self contained three-dimensional (3D) nanoscale magnetic resonance imaging system enabling microscopy and structure determination at the single molecule level. Rather than rely on large external field gradients for spatio-frequency encoding, which require precise characterisation, we instead use the natural spin-dipole field of the probe spin to provide highly stable spatial encoding of target nuclear frequencies. The protocol interleaves three carefully constructed levels of quantum control: decoupling of nuclear spins in the target molecule, frequency selective driving of target spins in dipole slices formed by the probe's natural gradient field, and signal accumulation on the probe spin from which nuclear spin density and 3D molecular structure can be determined. To be useful, any single-molecule sensing system must be able to match or exceed the resolution and precision of established ensemble techniques, the state of the art of which are currently at the angstrom level^23^,^24^,^25^,^26^,^27^,^28^,^29^,^30^,^31^. Using the 1kDa rapamycin molecule (National Center for Biotechnology Information, PubChem Compound Database, CID=5284616) as a non-trivial test case, we simulate the nano-MRI protocol—nuclear spin decoupling, frequency space selective addressing, data acquisition and analysis, spin-density image formation and molecular reconstruction. We obtain 3D images of the hydrogen nuclear spin density (an important distinction to conventional X-ray crystallography where it is difficult to obtain signal from ^1^H nuclei) and with isotopic labelling we show it is possible to image ^13^C atomic sites. Under currently accessible experimental conditions the average image resolution achieved is at the angstrom level, with the associated reconstruction error at the sub-angstrom level. Further optimization could incorporate higher order detection sequences, adaptive sampling techniques, and/or pre-exploration using fast coarse grained information about a molecule's structure. Specific information about molecular conformation or similar mapping applications could be obtained relatively quickly using distinct nuclear spin labels^32^. Importantly, the quantum probe MRI technique does not require complex sample preparation, and is ideally suited to the challenging *in situ* membrane protein problem. The NV system is an obvious candidate for implementation, particularly for fast coarse structure determination and/or labelled conformation dynamics under ambient conditions. However, it is inevitable that molecular motion will compromise resolution for precise structure determination—for these applications the target molecule will need to be immobilized as far as possible, for example by operating at low temperatures, and snap freezing the target with its associated hydrated environment^33^,^34^,^35^. Such a prospect also opens up the possibility of exploiting the extremely long coherence times of phosphorus donors in silicon (Si:P)^36^,^37^, and associated single-atom fabrication techniques^38^, for high-precision molecular imaging.

## Results

### System overview

**Figure 1:**
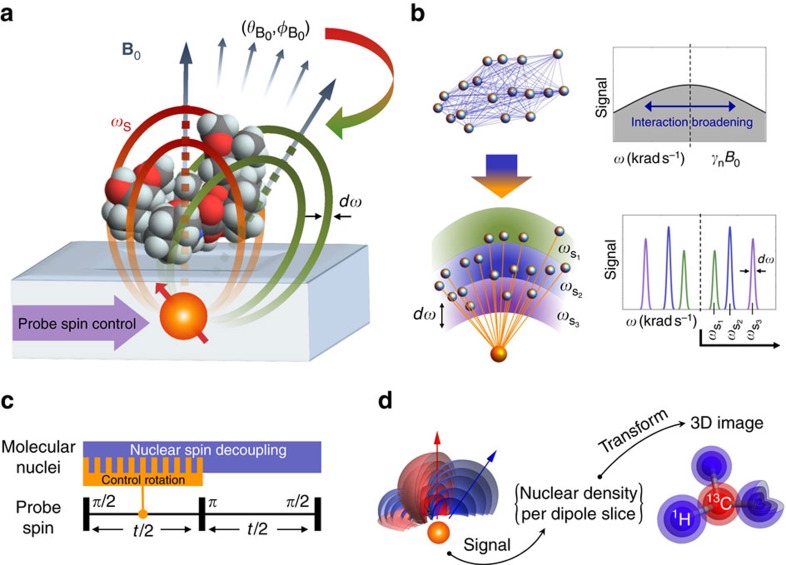
Overview of single-molecule MRI using a quantum spin probe. (**a**) The set-up consists of a controllable electronic probe situated 2–4 nm below the surface of the substrate, above which a molecule is positioned. The probe acts as both sensor and gradient field source for the spatial-frequency encoding of the nuclear spins in the target molecule. The equipotential slices (frequency, *ω*_S_) of the probe's coupling field gradient have a characteristic dipole–dipole lobe shape, and can be spatially rotated by varying the direction (*θ*_B_0__, *ϕ*_B_0__) of the background magnetic field **B**_0_ (magnitude of order 1-2 T). (**b**) Initially, the NMR spectrum of the molecule is broadened by the numerous nuclear dipole–dipole interactions (blue lines). The protocol decouples nuclei from each other in the presence of the coupling gradient field of the probe, and resonant excitation of target spins at *ω*_S_ provides a spatial MRI signal encoded on the probe state. In the spectrum obtained by sweeping the excitation frequency, the peak amplitudes characterize the spin density over the corresponding probe coupling slice. (**c**) High-level schematic of the interleaved control protocol structure consisting of a spin-echo sequence on the probe spin, and the slice-selective controlled nuclear spin rotation (excitation) embedded into a nuclear decoupling sequence. (**d**)The target molecule's nuclear density is sampled for multiple orientations of the interaction slices, followed by transformation from dipolar-slice space to cartesian space, to produce a 3D nuclear spin density image of ^1^H atoms, or other non-zero nuclear spin species such as ^13^C. Atomic positions are directly extracted from the density image data.

The set-up is shown in Fig. 1a and consists of a single controllable electronic spin defect in a semiconductor lattice, located 2–4 nm below the substrate surface, above which the target molecule is located. For high-resolution imaging we assume the vibrational and rotational degrees of freedom in the molecule are suppressed, by keeping the system and its hydrated environment in a typical temperature range of 77 K and below^34^. Positioning the molecule above the spin probe can be achieved in several ways. Mechanical placement of the molecule onto a silicon surface can be performed using an STM/AFM tip, or with optional initialisation of covalent bonding via STM current^39^. Alternatively, a number of molecules can be placed on the surface statistically ensuring probe to target proximity^40^. The spatial localization of the probe spin allows it to be in close proximity to the molecule and the dipole interaction field between the molecular nuclear spins and the electronic probe spin assumes the role equivalent to the classical gradient field in conventional MRI (Fig. 1a). The direction of the background magnetic field **B**_0_ (with magnitude of order 1-2 T), defines the quantization axis of both the probe spin and the nuclear spins of the molecule, thus providing a way of controlling the effective strength and direction of the dipole interaction between the probe and nuclei spins. The nuclear spins in the molecule are subjected to two different regimes of control—a high amplitude constant radio frequency field used for nuclear decoupling, and a considerably weaker NMR field for resonant excitation. We primarily focus on imaging the ^1^H nuclear density as the most abundant species in organic molecules, however, as we will show the method is also applicable to other spin species (for example, ^13^C or ^14,15^N) through isotope enrichment. As a MRI based method, our protocol encodes structural spatial information onto frequency space, however, in contrast to conventional MRI, where the magnetic field gradient forms equipotential planar slices, the nuclei experiencing equal interaction with the probe spin are located on curved, non-uniform slices, corresponding to dipole lobes (Fig. 1a). The exact shape of the interaction slices depends on the probe's electronic wave function, which for simplicity we assume is highly localized (for example, NV), but in principle can be pre-characterized (for example, Si:P)^41^ and/or calibrated using known structures. Details of the actual probe electronic wave function would modify the coupling profile, however, the overall imaging methodology itself is independent of any particular shape of the interaction slices.

The NMR spectrum of a stationary molecule is dominated by its intrinsic dipole–dipole iterations (Fig. 1b, top), causing each nucleus to be detuned from the resonant frequency defined by the probe interaction slices. The average extent of such detuning for ^1^H nuclear spins is comparable to their coupling to the probe spin, leading to a full loss of correlation in mapping from real to frequency space. To overcome this obstacle, the control protocol simultaneously suppresses the coupling between the nuclei themselves, and extracts the information about the number of nuclei present in each of the interacting slices (Fig. 1b, lower). In the first instance, we consider well-established pulse sequences in the solid state NMR and quantum sensing literature as foundations for the control protocol. At a high level, the protocol (shown in Fig. 1c) is composed of a spin-echo sequence (*t*/2−*π*−*t*/2) of length *t* performed on the probe and a decoupling sequence applied on the nuclear spins. In the first time period (0→*t*/2), controlled rotations of the nuclear spins located at a chosen resonant interaction slice are performed based on the probe spin state. These operations induce a phase in the probe spin state which accumulates information about the nuclear number density present on the probed interaction slice. In the second time period (*t*/2→*t*) the nuclear spins are subjected to a decoupling sequence only, inducing no additional phase in the probe spin state. This particular form, where only one half of the probe's spin-echo sequence receives the information, is chosen in particular for its high quality signal output of a simple sinusoidal form. In principle, multi pulse dynamical decoupling sequences (for example, CPMG, XY8, UDD (ref. 42)) can be used on the probe, containing multiple appropriately implemented control rotation segments. However, a balance is required; as the rate of information transfer from molecular nuclear spins to the probe is set by the coupling strength between the two, high order sequences can have a negative effect on sensitivity as they serve to decouple the probe from the nuclear spins.

### Interleaved quantum control and measurement protocol

**Figure 2:**
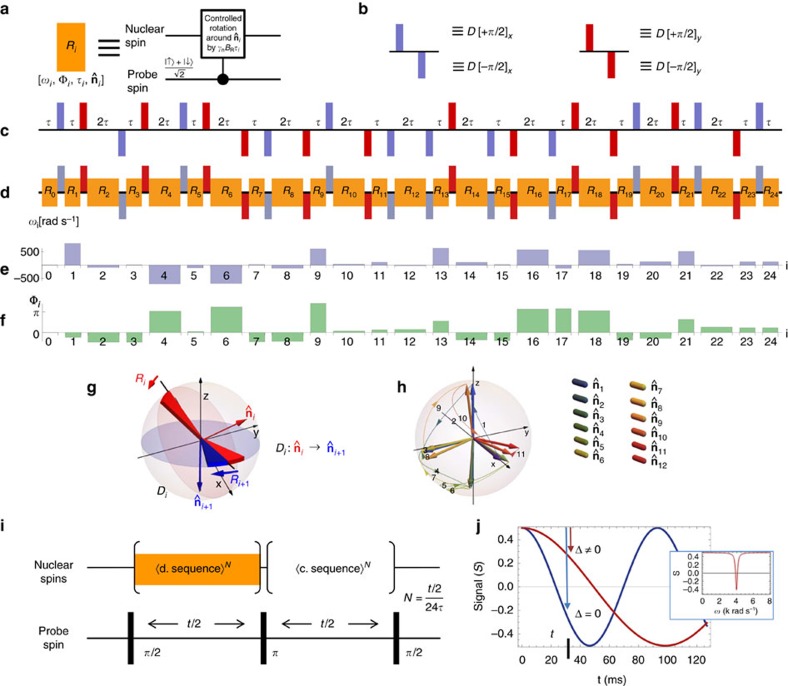
Detailed outline of the proposed control protocol. (**a**) The *i*th control rotation pulse, *R*_*i*_, of length *τ*, selectively rotates the population state of the nuclear spins located at a particular coupling slice of resonant frequency *ω*_S_. (**b**) The non-selective, spectrally broad, 90° pulses 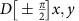 used for the nuclear decoupling. (**c**) The nuclear decoupling sequence (based on CORY24) has no effect on the probe spin as the nuclear pulse frequency is well away from the electronic resonant condition. (**d**) The control rotation pulses *R*_*i*_ are embedded into the decoupling sequence in place of the free precession times. Their phase and frequency is specifically tailored to produce a cumulative resonant nuclear spin state rotation in the selected interaction slice. (**e**) Example of the frequency detuning control of the polar tilt of *R*_*i*_ rotation axis (initial 24 pulses for a nuclear spin in Γ_S_=8 × 10^3^ rad s^−1^ interaction slice). (**f**) Associated phase of the driving field required for the *R*_*i*_ pulses in **e**. (**g**) The phase and frequency of *R*_*i*_ pulses synchronised through tracking the plane of rotation (direction of the driving field) as it is transformed by successive *D* pulse. (**h**) Example of several consecutive planes of rotation defined by their normals 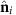. Each interaction slice has a unique pattern of driving field parameters (phase and frequency) as the decoupling pulses are timed according to the background magnetic field from which the interaction slices are frequency shifted by the coupling to the probe spin. (**i**) High-level schematic of the total control protocol. The phase induced in the evolution of the probe spin by the selective control rotation of the nuclear spins is a function of the number of nuclear spins in the probed interaction slice. The *π* pulse on the probe serves to refocus both the probe phase, and the effects of probe's gradient coupling on the nuclear spins. (**j**) The temporal evolution of the sequence on/away from resonance (blue/red). The signal is obtained by choosing an observation time *t* and sweeping the frequency *ω*_S_.

The multi-tier control sequence is shown in more detail in Fig. 2. The notation for probe-target operations used in writing out the entire sequence is defined in Fig. 2a and b. The *R* pulses (Fig. 2a) are resonant to a particular slice and transfer target spin information to the probe. The strong 90° non-selective decoupling radio frequency pulses, 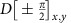, of field strength *B*_D_ (Fig. 2b), suppress the coupling between the nuclei. For definiteness, in this initial proposal we employ the CORY24 decoupling sequence used in conventional NMR (ref. 43), as shown in Fig. 2c (Methods section, homo-nuclear decoupling). A single segment of the overall sequence, interleaving probe-controlled rotations, *R*, and nuclear decoupling pulses, *D*, is shown in Fig. 2d. The set of pulses {*R*_*i*_(*ω*_*i*_,Φ_*i*_,*τ*_*i*_,*B*_R_)__*i*__}, (Fig. 2a) are applied during the free precession time *τ*_*i*_{*τ*, 2*τ*} around a carefully designed set of axes, {_*i*_}, which are determined by the driving field amplitude *B*_R_, frequencies {*ω*_*i*_}, and phases {Φ_*i*_} (shown in Fig. 2e and f; the construction is described in more detail below). Each *R_i_* pulse induces a perturbation in the states of a selected nuclear population, in a given dipole slice, of magnitude proportional to *γ*_n_*B*_R_*τ*_*i*_, where *γ*_n_ is the target nuclear gyromagnetic parameter. Critically, the consecutive *R* pulses are synchronised to produce a cumulative signal, corresponding to the number of nuclear spins targeted, on the probe spin. This is achieved by setting the parameters *ω*_*i*_ and Φ_*i*_ in accordance with both the previous *R*_*i*−1_ pulse and decoupling pulses, *D*. The overall nuclear dynamics are characterized by the slow modulation envelope due to *R* pulses on top of the fast dynamics of the decoupling sequence. Under a relatively long spin-echo sequence (*t*∼1/*γ*_n_*B*_R_≫*τ*), the probe is only sensitive to the slow dynamics associated with the nuclear spins located at the selected interaction slice forming a final signal corresponding to the number of target spins in that slice.

We now analyse the resonant slice conditions in more detail. Consider a nuclear spin at a given coordinate **r** (in the lab frame) relative to a well-localized electronic 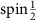 probe, under a magnetic field **B**_0_=*B*_0_(sin*θ*_B_0__ cos*ϕ*_B_0__, sin*θ*_B_0__ sin*ϕ*_B_0__, cos*θ*_B_0__) of constant magnitude *B*_0_ and variable orientation (*θ*_B_0__,*ϕ*_B_0__). The nuclear spin resides in the interaction slice of equi-coupling strength Γ_S_(*θ*_B_0__, *ϕ*_B_0__;**r**) as defined by the secular dipole–dipole coupling field:


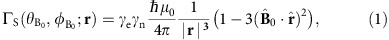


where the 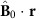 term defines the orientation of dipole interaction slices as function of background magnetic field direction (*θ*_B_0__,*ϕ*_B_0__). The decoupling pulses *D* are timed with respect to the background magnetic field frequency *γ*_n_*B*_0_, however, the nuclear spins precess with frequencies according to the dipole slice resonant coupling, that is, *ω*_S_^(±)^=*γ*_n_*B*_0_±Γ_S_/2 (for clarity we suppress angular dependencies when not required), where *ω*_S_^(±)^ represents the resonant nuclear frequencies associated with each of the probe's two spin states. Therefore, as observed in the frame of an interaction slice (defined by Γ_S_), the axis of the decoupling pulses rotates with relative frequency Γ_S_/2. The plane defined by a normal in which *R*_*i*_ induces a rotation thus transforms into a new plane 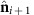 under the influence of a decoupling pulse, *D*_*i*_. To achieve a cumulative phase signal on the probe, the *R*_*i*+1_ performs a control rotation in the transformed plane 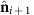 to add to the previous rotation of the nuclear spin state (Fig. 2g). The initial pulse, *R*_0_, constitutes a controlled rotation in the direction _0_, which can be an equatorial direction of choice. At the end of the CORY24 sequence the net cumulative control rotation of the selected nuclear spins is returned back to this initial direction, leading to a net change in the z-projection of the nuclear spin states which is detected by the probe. Unless explicitly stated otherwise, from here on we assume the interrogation of the spectrum is performed in the positive frequency side 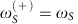, omitting the superscript (as illustrated in Fig. 1b). Relative to the initial axis _0_ any subsequent control rotation *R*_*i*_ is formulated by transformation of _0_ under the decoupling *D* pulses as observed in the frame of the particular interaction slice characterized by coupling strength Γ_S_:


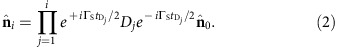


where *D*_*j*_ is the *j*th decoupling pulse occurring at time *t*_D*_j_*_. Once the unique sequence of driving directions is determined for a given target interaction slice (Fig. 2h), it follows that the pulse *R*_*i*_ resonant with the interaction slice of coupling strength Γ_S_, is constructed by applying the magnetic field of frequency *ω*_*i*_ and initial phase Φ_*i*_ as defined by 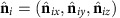:


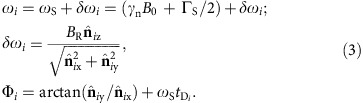


The frequency shift *δω*_*i*_ defines the polar tilt of the rotation axis while the phase Φ_*i*_ defines the azimuthal direction. The frequency and phase control are depicted in Fig. 2e,f. We note that from the perspective of practical implementation, the phase of the driving field of each pulse *R*_*i*_ is more crucial than the frequency shift *δω*_*i*_. In the case of high background magnetic fields (*B*_0_∼1T) and relatively low driving fields (*B*_R_∼1 μT) the frequency shifts are relatively small. Furthermore, the average *δω*_*i*_ is equal to zero. Thus, keeping the driving frequency constant (unlike the phase) *ω*_*i*_→*ω*_S_ across all *R* pulses produces the resonant behaviour comparable, to within several percent, to the full variable frequency control. The fully integrated control protocol is depicted in Fig. 2i.

When the power broadening due to the fine driving field *B*_R_ is smaller than the probe coupling Γ_S_, and the probe spin is held in an equal superposition state, the signal *S*_*α*_ (Fig. 2j) generated by the *α*th nuclear spin under the sequence is well approximated by the following form:


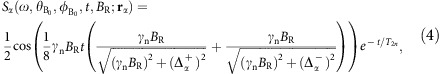



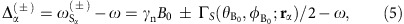


where **r**_*α*_ is the coordinate of the of the *α*th nuclear spin, (*θ*_B_0__,*ϕ*_B_0__) is the direction of the background magnetic field, *T*_2n_ is the effective transverse coherence time of nuclear spins, and 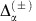 is detuning of the scanning frequency *ω* from the resonance frequencies, 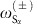. The typical temporal dependence of the signal (Fig. 2j) has a sinusoidal shape, analogous to Rabi oscillations, reflecting the cumulative effect induced by fine driving *R* pulses on the nuclear spin state. In addition to providing a high quality signal, the presence of selective, slow nuclear evolution in only one half of the probe spin-echo sequence results in a characteristic detuning behaviour. As 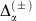 increases, the frequency of the signal decreases, while the maximum amplitude remains unchanged, as illustrated by Fig. 2j. This is particularly useful for the generation of a clean signal trace as a function of sweep frequency, *ω*, at a fixed time *t*, providing an effective Lorentzian signal peak. For well decoupled nuclear spins (*T*_2n_>*t*) in an equally mixed spin state, the total signal from a slice resonantly addressed by frequency *ω* can be expressed as a product of signals originating from each individual nucleus:


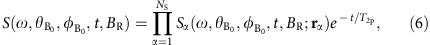


where *N*_S_ is the total number of spins in the slice of the species, and *T*_2p_ is the transverse coherence time of the probe spin. We emphasise that the outlined methodology can be generalized with respect to variations in the nuclear decoupling and probe detection sequences. In the case of decoupling sequences that have less or no free procession periods, the control rotation, *R*, pulses can be applied simultaneously with any decoupling pulse, provided the two commute. An additional feature of the control sequence is the positive effect on the probe's coherence time *T*_2p_. By suppressing the dipole coupling between the nuclei, the protocol simultaneously modifies the intrinsic magnetic fluctuation spectra of the molecule, and thus its frequency spread is reduced to a narrow band around a single high frequency (1/*τ*) that is outside the detection range of the probe sensing protocol. This property opens up a noise-free window in low-frequency spectrum, which is particularly suitable for high-resolution detection of remote nuclear spins.

**Figure 3:**
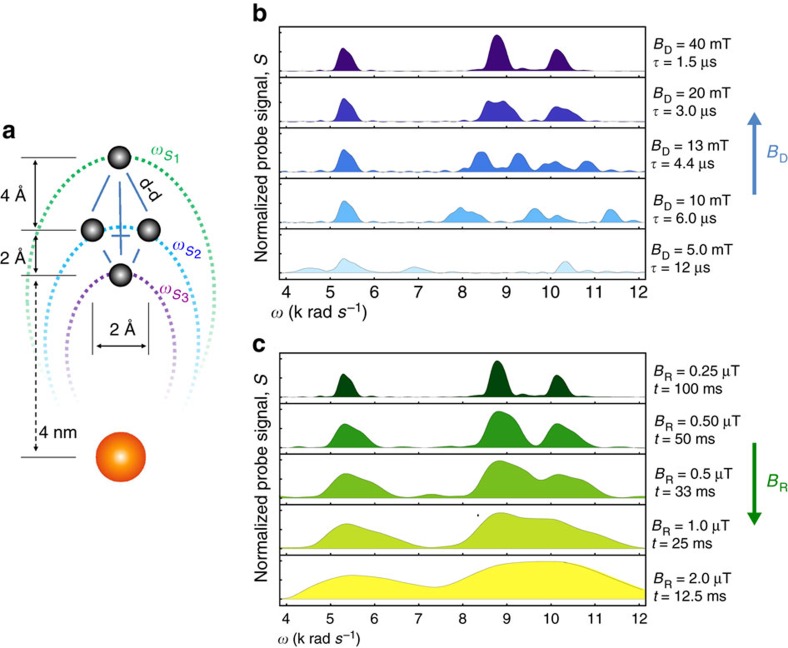
Quantum simulation of the target nuclear spin decoupling sequence. (**a**) Four ^1^H nuclei (grey), positioned 2 Å apart, located 4 nm above the probe electronic spin (orange). The spatial interaction slices are shown, with labelled resonant frequencies *ω*_S1→3_. (**b**) The MRI spectra (for each plot the probe signal is normalized to unity (0→1), where *S*=0 represents the mixed state and *S*=1 is the maximum z-projection of the probe spin state) as a function of decoupling field strength *B*_D_, for an order of magnitude ratio between the decoupling pulse (*D*[*π*/2]) length and time between the pulses *τ*, 
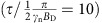
. The peaks at frequencies {*ω*_S_1__,*ω*_S_2__,*ω*_S_3__} in the upper plot (decoupled spins) correspond to the nuclear density in the associated interaction slice. The panes moving downward show the loss of spatial-frequency coding as the decoupling efficiency decreases. (**c**) The MRI spectra (same normalization convention as in **b**) under different driving strength fields *B*_R_ exhibits typical power broadening behaviour, depicted, for visual clarity, in the spin decoupled regime *B*_D_=40 mT with sampling times *t* adjusted to preserve the signal normalization (*γ*_n_*B*_R_*t*=constant). The simulations were performed at *B*_0_=2 T and *T*_2p_=50 ms.

To illustrate the behaviour of the integrated detection protocol (Fig. 2i), we perform a fully quantum simulation of a cluster of ^1^H nuclei at realistic distances from the probe which couple through their mutual dipole–dipole interactions (Fig. 3a). In the spectrum obtained by sweeping the frequency, *ω*, the peak heights contain information on the number of nuclei located in the same interaction slice (Fig. 3b, front plane). Figure 3b depicts the spatio-frequency encoding behaviour of the MRI spectrum as a function of the detection protocol's decoupling parameters. The upper pane (Fig. 3b) corresponds to the strong decoupling regime. Moving down towards the lower pane corresponds to weaker decoupling where the spatial encoding of the MRI spectrum is distorted due to emerging dipole–dipole interactions between the nuclei, reflected in progressive splitting of initial MRI peaks. The results indicate that decoupling fields *B*_D_ with amplitudes of order 20–40 mT are required. This estimate is appropriate in the case of more complex nuclear systems, as the local environment of nuclear spins dominates the coupling magnitudes. Figure 3c depicts the degree of power broadening behaviour of the detection sequence as the fine driving field strength *B*_R_ increases, indicating minimum quantum probe coherence times *T*_2p_ requirements in the range of 30–50 ms for sufficient resolution of molecular features at the angstrom level. This is well within reach of coherence times for P donors in purified silicon at a typical operational temperature of order 100 mK (refs 36, 37). The coherence times of the NV centre in diamond are at present insufficient for full-structure determination, although the required regime may, in principle, be reached by further diamond surface engineering and isotropic purification related to nitrogen and carbon(13) baths^44^,^45^. Current experimental coherence times for near-surface NV centres may allow conformation detection based on imaging the locations of a small set of spin-labels.

### MRI measurement and structure determination

**Figure 4:**
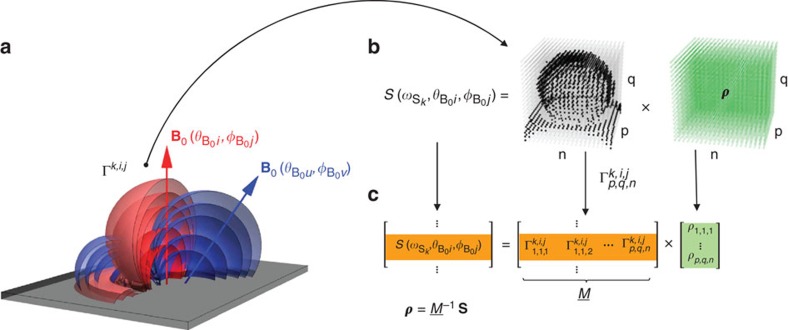
Protocol for nuclear 3D density imaging: (**a**) Illustration of interaction slices Γ^*k*,*i*,*j*^ resonantly addressed by *ω*_S*i*_ at various orientations (*θ*_B_0_*i*_,*ϕ*_B_0_*j*_) showing the characteristic dipole–dipole shape of the coupling equipotential (Γ_S_(*θ*_B_0_*i*_,*ϕ*_B_0_*j*_; **r**)=*ω*_S*_i_*_). (**b**) An example of data decomposition for a single slice where the volume containing the target molecule is discretized (*p* × *q* × *n*) allowing for voxelated representation of both the nuclear density and each interaction slice. (**c**) The vectorized form of the voxelated nuclear density ***ρ***, the voxelated interaction slices 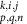 and the vectorized form of the measured signals **S** taken for all orientation and fervency values, enable construction of a map 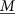 from the nuclear density *ρ* in cartesian space to the measured signal **S** in slice-space. Each row of the map 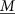 corresponds to a vector form of a single voxelated interaction slice 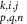. This representation allows for inversion of the map 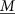 leading to extraction of the 3D nuclear density 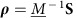.

**Figure 5:**
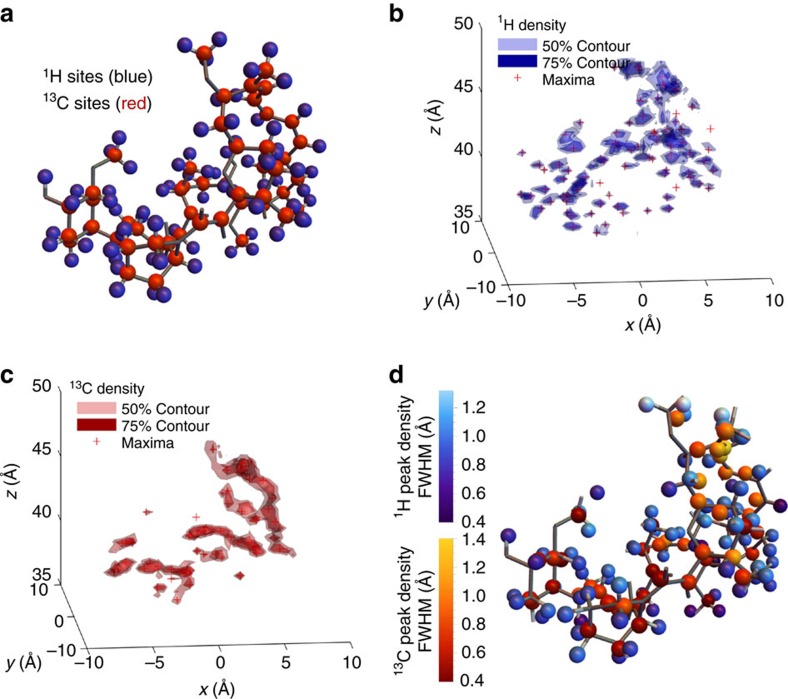
Simulation of the nano-MRI protocol carried out on a single rapamycin molecule (C_51_H_79_NO_13_). (**a**) Hydrogen and carbon substructure of the rapamycin molecule. Sampling was performed over 150 slices for each of 11 × 24 latitude and longitude orientations of **B**_0_ with *M*_S_=2000 measurements per slice and sequence length *t*=8 ms. (**b**,**c**) Extracted ^1^H and ^13^C nuclear densities, respectively. The 3D density is represented through 50% and 75% contours that reveal subthreshold features. Atomic positions are identified corresponding to local maxima in the 3D density that are >90% or greater in relative terms (shown as crosses in (**c**,**d**)). The uncertainty in position (effective resolution) is estimated from the average of the (*x*,*y*,*z*) FWHM cross-sections of the 3D density peaks. Peaks closer to the probe (smaller *z* values) are narrower due to stronger gradient coupling to the probe, while isolated peaks are further localized due to a greater relative contrast in the numerical inversion procedure. (**d**) Reconstruction of the atomic positions and associated resolution (colour scales) are shown for ^1^H (blue colour scale) and ^13^C (red colour scale). The sphere size corresponds to the average statistical deviation in comparison to the exact coordinates of Δ*r*=0.5 Å (superimposed on the data is the exact skeletal structure from **a**.

We now illustrate the entire protocol using a non-trivial example—the rapamycin molecule (C_51_H_79_NO_13_). This particular molecule, in the mass range ∼1 kDa, is around three orders of magnitude larger than that considered in proposals for 1D and 2D NMR spectroscopic approaches^18^,^19^,^22^. In our approach, once a sufficient level of decoupling is assumed, it is possible to simulate the entire structure determination protocol. We apply the sequence to acquire ^1^H and ^13^C nuclear density data and obtain the molecular coordinates for the two atomic species. The spectral data was sampled at multiple orientations of the background magnetic field as depicted in Fig. 4a (Methods section, 3D imaging protocol), and contains the nuclear density of the target molecule in dipolar-slice space—numerical inversion into cartesian space (Fig. 4b,c) yields the molecular nuclear 3D densities shown in Fig. 5b,c. The ^1^H and ^13^C atomic positions in the rapamycin molecule are readily observable by inspection of contours of 50 and 75% contrast around 3D density peaks having distinct spherical shapes. The atomic coordinates are reconstructed by thresholding local maxima in the nuclear densities above 90% (crosses, Fig. 5b,c), and estimating the uncertainty in the atomic positions by average of the full width at half maximum (FWHM) in three axial cross-sections of the 3D density peaks. Figure 5d shows the atomic site coordinates with the colour scale conveying the spatial uncertainties (corresponding to the widths of the density peaks) in atomic positions ranging from 0.4 Å to 1.4 Å. We note that these coordinates are derived directly from the density image, without potential energy minimization or other post-processing. Comparison of the reconstructed atomic coordinates to the true molecular coordinates, gives an average statistical variation of 0.5 Å, represented as the radius of the spheres in Fig. 5d. Using this simple data collection strategy, the total acquisition time amounts to ∼175 h under realistic experimental conditions to image the ^1^H structure of rapamycin (and twice as long for the ^13^C structure). These calculations assumed probe and nuclear target coherence parameters *T*_2p_=50 ms, *T*_2n_=50 ms and a detection sequence of total length *t*=8 ms, with shot noise limit given by *M*_S_=2000 single shot measurements per slice over 150 slices for each of the 264 **B**_0_ orientations (for details see Methods section, parameters for the molecule example). With the outlined parameters, the protocol provides a hemispherical imaging volume of ∼8 nm in radial extent above the probe–sufficient for imaging proteins across cell membranes (∼4 nm thickness). Where larger imaging volumes are required, it may be possible to augment the protocol with additional external (classical) gradient fields^21^, however, with increasing probe-target distance detection is more limited. The variation in 3D density resolution along the *z* axis is consistent with the simple constant sampling methodology used in this example, where the frequency sweep steps are constant yet the dipole strength gradient is not. Thus, the results from this rudimentary sampling procedure should serve as an indicator of the upper limit on the total acquisition time and resolution achievable.

The rapamycin example illustrates the potential of full atomic scale imaging of single molecules based on naturally abundant nuclei, such as highly dense ^1^H atoms that detail the structure of organic molecules. However, for applications where only limited information is required (for example, conformation and/or ligand state/position), targeting characteristic nuclear spin labels (that is, ^14,15^N, ^13^C, ^31^P) 1–2 nm apart, can be achieved up to two orders of magnitude faster compared with full atomic scale imaging. This is a consequence of order of magnitude reductions in both the required number of interaction slices and the measurement sequence length *t*. In this case, the requirements on the probe's coherence times decrease to the 3–5 ms range, with further 2–3 orders of magnitude reduction in field strength needed for nuclear decoupling pulses. This opens up experimental applications in room temperature individual molecule conformational mapping based on NV centre in diamond, particularly for conformation detection based on electron spin labels^46^ for which there is an additional three orders of magnitude speed up due to the stronger magnetic moment. In this case the probe's transverse coherence time is required to be in the microsecond range or greater which is commensurate with current values for near-surface NV centres^11^,^40^,^47^.

## Discussion

In conclusion, we have introduced a new concept for a nano-MRI molecular microscope system based on a generic quantum spin probe, and showed by direct quantum simulation that it allows for the determination of single-molecule structure to high resolution. In this approach, the quantum probe acts as both MRI sensor and gradient field, encoding the target's real-space spin density in frequency space. The key to the system is a carefully designed protocol interleaving nuclear spin decoupling and phase accumulation on the probe. By systematically performing measurements on the quantum probe over many positions and orientations of the probe-target interaction slices, the probe acquires information about the nuclear spin density in the target molecule. After the deconvolution procedure the nuclear spin density can be mapped out and the atomic coordinates determined. The technique was tested on a non-trivial example—the rapamycin molecule—using a rudimentary sampling procedure and quantum probe coherence parameters already achieved experimentally for phosphorus spin qubits in silicon^36^,^37^. The resolution and average structural errors were found to be at the angstrom level and below. Further improvements and optimization are expected to greatly reduce the overall acquisition time which here should be considered as an upper limit. The protocol length, frequency steps and fine driving field can be refined through a nonlinear rate of change of the probe interaction field, leading to more efficient molecular imaging with uniform resolution through the sampled volume. Our imaging method is suitable for molecules considerably larger than the presented example. Targets of >20 kDa would benefit significantly from adaptive sampling, that is, starting from a fast low resolution density estimate and iteratively modifying the sampling parameters together with orientation of the interaction slices. For aggregate protein structures of significant spatial extent (10 s of nanometers), an addition of an external classical gradient field (for example, a magnetic tip) could provide a pathway to remote spatio-frequency encoding. However, more work needs to be done to demonstrate the level of decoupling achievable in the classical gradient fields, experimental timescales required to detect variations of the nuclear spin density at such distances, and the additional influence of the gradient on the quantization axis of both the target and probe spins. The use of more effective decoupling pulses could also have significant impact on the efficiently of the total detection protocol. The protocol was presented for the case of a general spin probe, however, the experimental parameters considered are consistent with current coherence measurements of phosphorus qubits in silicon (Si:P). In the case of the NV spin one could implement a pulsed background field, or attach the molecular sample to an AFM tip scanned through the static field, to avoid off-axis contrast loss, in addition to engineering both appropriate surface and isotopic properties of diamond required to achieve higher electron spin coherence times. The detection protocol outlined here utilises only a single electronic spin and, as such, it is limited by the transverse coherence time of the probe *T*_2p_. An additional storage qubit, such as the nuclear spin available in both NV and Si:P implementations, can be utilised during the control rotation phase by swapping signal information on the electron spin to the nuclear spin^48^,^49^,^50^,^51^ to significantly extend the detection limit to the probe's longitudinal *T*_1p_ coherence time. With straightforward sample preparation of molecular targets in their *in situ* environments, for example, hydrated and coupled protein-drug systems, and hybrid options for adding extra sources of gradient fields (for example, by site-directed spin labelling) to scale up to larger biomolecules, quantum probe-based MRI has significant potential for true single-molecule microscopy.

## Methods

### Homo-nuclear decoupling

Suppression of coupling between nuclear spins is routinely performed in solid state NMR, by physical or Hilbert-space rotations^52^. Physical rotations around the dipole–dipole magic-angle axis have proved useful in practice, however, nanometer scale rotations of a single molecule relative to a single spin probe are outside the reach of immediate technology, thus we propose Hilbert-space techniques to achieve the same result. Out of multiple families of Hilbert-space approaches, the two dominate branches in the group of on-resonance pulses are the solid-echo and magic-echo^27^,^52^,^53^ based sequences. The former utilise multi-axis 90° pulses to perform effective spin rotation about the magic-angle axis, and the latter are based on 360° pulses. Without loss of generality, we consider the solid-echo type sequences and show how they can be integrated with control rotations on the nuclear spins. These decoupling sequences are made up of unit blocks of the following form: 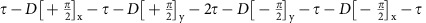 where 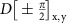 are 90° square pulses around positive/negative *x* or *y* axis, and *τ* is the free evolution time of appropriate duration^53^.

Hilbert space decoupling approaches are designed for conventional NMR techniques under uniform background magnetic fields, therefore their application in a gradient field regime requires detailed consideration. A dipole–dipole NMR spectrum of a molecule in a uniform magnetic field **B**_0_ is symmetric around the frequency set by the background field *γ*_n_*B*_0_, where *γ*_n_ is the nuclear gyromagnetic ratio. The square decoupling pulses are also centred around the same frequency, but have a spectral spread wider than the dipolar molecular NMR spectrum. This ensures appropriate narrowing of the spectrum towards the background frequency *γ*_n_*B*_0_. In contrast to classical gradient fields, we can choose to maintain this symmetry of the NMR spectrum by keeping the probe spin in an equal superposition throughout application of the decoupling pulses, while keeping the free precession time equal to the full number of background field periods *τ*=*n*_d_2*π*/*γ*_n_*B*_0_, *n*_d_∈. The probe itself does not participate in the effective magic-angle spinning as its electronic spin has different resonant frequency. In turn the probe coupling terms to each of the nuclei, Γ_S_(*θ*_B_0__, *ϕ*_B_0__;**r**), remain unaffected and define the interaction slices characterized by polar direction (*θ*_B_0__, *ϕ*_B_0__) of the background magnetic field **B**_0_. This feature allows the spin-echo sequence on the probe to refocus the phase offsets encountered by nuclear spins on different interaction slices, thus maintaining their coherence under the gradient coupling regime.

### 3D imaging protocol

This section details the general steps leading to the generation of the 3D spatial image of the molecular nuclear density. The primary sampling is done by sweeping the fine driving frequency *ω* in discrete steps of *dω* from *ω*^m*in*^ to *ω*^m*ax*^. The upper frequency limit *ω*^m*ax*^ is set by the value of the interaction at the substrate surface, immediately below the molecule. The lower frequency bound *ω*^m*in*^ must be no greater than the minimal coupling experienced in the molecule, spatially associated with the uppermost part of the molecule.

Relative to the probe, the physical shape of a resonant interaction slice is defined by a equipotential of the coupling field between the probe and the nuclei having value equal to *ω*. In frequency space, the spectral broadening of the signal generated by the power of the driving field *B*_R_ is set equal to the sampling step *dω*, ensuring sufficient spectral coverage. In real space, the dipole slice corresponds to a spatial region of non-uniform width, depending on the strength of the driving field *B*_R_ and the gradient of the coupling field.

The signal values generated by the frequency sweep contain relative information about the number of nuclear spins located on each of the interaction slices. The orientations of the interaction slices depends on the direction of the quantisation axis (Fig. 4). The frequency sampling is performed for multiple orientation of the quantization axis by reorienting the background magnetic field **B**_0_, in discrete latitude steps of *dθ*_B_0__ from *θ*_B_0__^min^ to *θ*_B_0__^max^ and azimuthal steps of *dϕ*_B_0__ from 0 to 2*π*.

We note that the signal for each resonant slice is a product of individual nuclear spin signals. This characteristic has to be considered both from sampling and from data processing perspectives. As a function of time, the signal approaches zero as the number of spins on a resonant slice increases. An appropriate choice of the fine driving strength *B*_R_ and the sequence length *t* has to be made. To yield optimal results this calibration can be optimised dynamically for a particular molecule of interest as part of the initial frequency sweep. Alternatively the sampling can be performed for several sequence lengths.

Given the signal exhibits a product structure, we devise a self-scaled derivative (SSD) approach to linearisation. We define the SSD, *S*_i_*(*x*) of a single signal function, *S*_i_*(*x*), with respect to a given parameter *x* as:


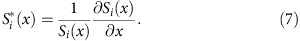


As the total signal is a product of signals of all *N* nuclei in the molecule, it follows that the SSD of the total signal with respect to a variable *x* transforms from product to linear structure:


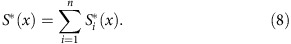


The data is linearized by computing the SSD for each frequency sweep with respect to the fine driving frequency *ω*. To obtain the MRI spectrum we perform a one-dimensional deconvolution to the frequency sweep data. The natural choice for the point spread function is the single nuclei spin response SSD function, *S*_i_*, symmetrically centred in the sweep range. The deconvolution step removes signal contributions of the spins on the neighbouring interaction slices, ensuring that the data linearly reflects the number of spins present at their corresponding slice.

To form the nuclear density image, the volume containing the molecule is discretized into *n* × *m* × *q* segments separated by a *dl*[Å] step. We construct a transform 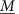 such that 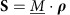 where ***ρ*** is a vectorized 3D nuclear density, and **S** is the equivalent form of the measured signal for all slice orientations. Each row of the matrix 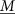 represents a discretised 3D map corresponding to a single interaction slice. The nuclear spin density is obtained by numerical inversion. This general approach is unconstrained by the shape of the resonate slices. From the 3D density image, atomic coordinates are extracted by observing the positions of the local maxima.

### Parameters for the the molecule example

For clarity, the method is demonstrated for a probe with a well-localized electronic spin, thus the interaction slices correspond to equipotential slices of the secular dipole–dipole coupling field:


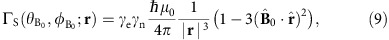



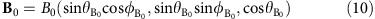


where **r** is a given nuclear coordinate vector relative to the probe in the lab frame and **B**_0_ is the constant magnitude background magnetic field controlled by polar direction parameters (*θ*_B_0__, *ϕ*_B_0__).

The parameters chosen for the simulation are as follows: the fine frequency sweeping range was *ω*^min^=100 rad s^−1^, *ω*^max^=15 krad s^−1^ with the sampling step of *dω*=100 rad s^−1^ (*ω*=*ω*_S_ is a resonance condition for probing nuclei in the slice *ω*_S_). The frequency sweeps were conducted for a range of orientations as given by latitude parameters 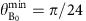, 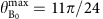, and 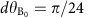 and longitude steps *dϕ*_B_0__=*π*/12. Each point was sampled at a fixed protocol length of *t*_H_=8 ms for ^1^H nuclei and *t*_C_=16 ms for ^13^C nuclei, with *M*_S_=2000 measurements per slice. The transverse coherence time of the probe and the effective coherence time of the nuclear spins were set to be respectively *T*_2p_=50 ms and *T*_2n_=50 ms. Such coherence times are already established for the Si:P system, and with suitable cooling and material improvements may be possible for the NV centre.

To obtain nuclear density images in 3D, the region around the molecule, *xε* (−1,1) nm, *yε*(−1,1) nm and *zε*(3.5,5) nm was discretized in cubic voxel of side length *dl*=0.5 Å, the inversion of the map 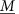 was performed by numerical least-square based approach^54^,^55^,^56^.

### Quantum simulations

Features of the numerical simulations (Fig. 3) performed to investigate the behaviour of the detection protocol (Fig. 2i) are characterized by the full quantum Hamiltonian, containing nuclear dipole–dipole interactions between individual nuclei and probe spins as well as between all nuclei themselves. This is a crucial requirement for accurate investigation of intricate dynamics between the homo-nuclear decoupling part of the detection sequence and the fine driving that transfers the relative spatio-frequency coded information about the nuclear spin density to the phase of the probe spin. The second requirement is for both the strong decoupling pulses as well as the fine driving pulses to be introduced, on the Hamiltonian level, as physical fields of appropriate amplitude shape and length. We model both of these pulse types as square in shape reflecting readily used experimental approaches as well as lowering computational requirements needed compared with Gaussian or Sinc shaped pulses that would in principle give better permanence to the total detection protocol. In contrast, unitary matrix operations are sufficient to capture the control of the electronic probe spin, since the required pulses are relatively fast with frequencies are well outside the nuclear spin range. The initial state of the nuclear target spins is dominated by high temperature regime due to strong dipole–dipole interactions, and can be well captured by mixed state density matrix representation. The coherence of the target nuclei depends strongly on their quantum interactions with one another, while the probe spin can be sufficiently well modelled through an additional explicit Lindblad term designed to model its decoherence properties unrelated to the presence of the molecular nuclei (such as a ^29^Si bath for Si:P probe, or ^13^C bath for NV-centre). Note, the nuclei-probe dipole–dipole interaction in conjunction with decoupling properties of the detection protocol automatically affects the coherence of the probe spin beyond the explicit Lindblad term.

### Data availability

All relevant data are available from the authors on request.

## Additional information

**How to cite this article:** Perunicic, V. S. *et al*. A quantum spin-probe molecular microscope. *Nat. Commun.* 7:12667 doi: 10.1038/ncomms12667 (2016).
